# Evaluation of approaches for identifying population informative markers from high density SNP Chips

**DOI:** 10.1186/1471-2156-12-45

**Published:** 2011-05-13

**Authors:** Samantha Wilkinson, Pamela Wiener, Alan L Archibald, Andy Law, Robert D Schnabel, Stephanie D McKay, Jeremy F Taylor, Rob Ogden

**Affiliations:** 1 The Roslin Institute and Royal (Dick) School of Veterinary Studies, University of Edinburgh, Easter Bush, Midlothian EH25 9RG, Scotland, UK; 2 Division of Animal Sciences, University of Missouri, Columbia, MO 65211, USA; 3 Wildgenes Laboratory, Royal Zoological Society of Scotland, Edinburgh EH12 6TS, Scotland, UK

## Abstract

**Background:**

Genetic markers can be used to identify and verify the origin of individuals. Motivation for the inference of ancestry ranges from conservation genetics to forensic analysis. High density assays featuring Single Nucleotide Polymorphism (SNP) markers can be exploited to create a reduced panel containing the most informative markers for these purposes. The objectives of this study were to evaluate methods of marker selection and determine the minimum number of markers from the BovineSNP50 BeadChip required to verify the origin of individuals in European cattle breeds. Delta, Wright's F_ST_, Weir & Cockerham's F_ST _and PCA methods for population differentiation were compared. The level of informativeness of each SNP was estimated from the breed specific allele frequencies. Individual assignment analysis was performed using the ranked informative markers. Stringency levels were applied by log-likelihood ratio to assess the confidence of the assignment test.

**Results:**

A 95% assignment success rate for the 384 individually genotyped animals was achieved with < 80, < 100, < 140 and < 200 SNP markers (with increasing stringency threshold levels) across all the examined methods for marker selection. No further gain in power of assignment was achieved by sampling in excess of 200 SNP markers. The marker selection method that required the lowest number of SNP markers to verify the animal's breed origin was Wright's F_ST _(60 to 140 SNPs depending on the chosen degree of confidence). Certain breeds required fewer markers (< 100) to achieve 100% assignment success. In contrast, closely related breeds require more markers (~200) to achieve > 95% assignment success. The power of assignment success, and therefore the number of SNP markers required, is dependent on the levels of genetic heterogeneity and pool of samples considered.

**Conclusions:**

While all SNP selection methods produced marker panels capable of breed identification, the power of assignment varied markedly among analysis methods. Thus, with effective exploration of available high density genetic markers, a diagnostic panel of highly informative markers can be produced.

## Background

The identification and verification of the origin of individuals is useful in a variety of biological contexts and the practical applications of individual assignment protocols are extensive [1-3]. Topical issues in population, conservation and evolutionary biology can benefit from the inference of ancestry of individuals. In an applied context, genetic identification can shed light on issues such as the contribution of source populations in mixed fisheries [3,4], meat traceability or brand authentication [5], translocated or migrant individuals [6], structure and levels of discrimination amongst populations [7,8], anthropological forensic investigations [2] and tracking the trade routes of illegally poached animals [3].

Where there is sufficient genetic heterogeneity amongst populations genetic markers can be used to identify and verify the origin of individuals [7]. Customarily, the genetic marker routinely used in individual assignment studies has been hypervariable microsatellite loci (e.g. [4,5,7]). However, with the advent of genome-wide analytical technologies, microsatellites are now being widely replaced by Single Nucleotide Polymorphism (SNP) markers (e.g., [9]). SNPs are increasingly favoured as population genetic markers because they are highly abundant and widespread in the genome, homoplasy is virtually absent, methods to discover markers are reliable and subsequent automated genotyping through assay design can be easily implemented [10,11]. Numerous SNPs have been identified in the genomes of domestic animals, for example, in the dog (> 2.5 million) [12], chicken (~ 2.8 million) [13] and cattle (> 2 million) [14]. This has led to the technological development of standard products commonly termed 'SNP Chips', which enable the rapid automated large-scale production of genomic data. SNP Chips are now commercially available for many animal species (e.g., sheep, [15]; pigs, [16]) including the Illumina Bovine50SNP BeadChip (Illumina Inc., San Diego, CA) for cattle [17,18].

These new resources are highly informative; the Bovine50SNP BeadChip has already been used in genetic studies investigating population genetic structure [19], mapping for marker assisted selection of economically important traits [20,21] and unravelling the patterns of signatures of selection [19,22].

Dense genome-wide data is valuable but is relatively costly to produce and time-consuming or computationally expensive to analyse; it is therefore often desirable to reduce the number of markers by screening and selecting according to their information content to create reduced panels for population genetic analyses [23,24]. Several statistical selection methods are available to determine which genetic markers contain the most information to discriminate among populations. The statistic, delta, which measures allele frequency differences, is commonly used in the field of human genetics to assess marker information content [25,26]. Bowcock *et al.*, [27] suggested that informative genetic markers may be identified using Wright's F_ST _[28] and its derivatives [29]. Principle Component Analysis (PCA) has also been more recently proposed as an alternative method to determine population informative SNP markers [24]. Other algorithms have been developed to optimize the combination of loci selected (e.g., BELS, [30] and references therein); however, these approaches are computationally intensive and their execution may be prohibitively slow with large datasets.

The objective of this study was to examine methods for selecting population informative SNP loci. To achieve this we set out to determine the minimum number of SNP markers from the Illumina Bovine50SNP BeadChip (Illumina Inc., San Diego, CA) that is required for individual genetic assignment to discriminate a set of European cattle breeds (Table 1). This was approached in a two-stage manner. First, several SNP selection methods were evaluated to determine the genetic information content of each SNP marker and markers were ranked by decreasing level of informativeness for each of the methods. Second, the likelihood of assigning individual genotypes to their known breed origin was estimated by cumulatively increasing the number of SNP markers, according to the ranked estimates of each SNP marker's informativeness for each selection method.

**Table 1:** Information on the breeds

	Breed	*N*	Animal resources of *N*	*n*	Purpose	Origin	Distribution	Sampling Locality
1	Angus - British	23	several Scottish farms; majority different sires	23	Beef	Scotland (UK)	Global	UK
2	Angus - American	6124	Registered bulls and steers	25	Beef	Scotland (UK)	Global	USA
3	Brown Swiss	74	24 HapMap^1 ^(3 trios); remaining no pedigree	24	Dairy	Switzerland	Alpine Europe, Americas	USA
4	Charolais	135	26 HapMap^1 ^(3 trios); remaining registered	25	Beef	France	France, USA, Brazil, RSA	USA
5	Finnish Ayrshire	444	215 unrelated; 17 paternal half-sib families with average of 13 progeny per sire	10	Dairy	Scotland (UK)	Global	Finland
6	Guernsey	23	21 HapMap^1^; remaining unrelated	21	Dairy	Island of Guernsey (UK)	USA, UK, Oceania, RSA	UK
7	Hereford	143	32 HapMap^1 ^(4 trios); remaining registered	25	Beef	UK	Global	USA
8	Holstein	18904	Registered	25	Dairy	Netherlands	Global	USA
9	Jersey	93	28 HapMap^1 ^(3 trios); remaining registered	28	Dairy	Island of Jersey (UK)	Global	USA
10	Limousin	1621	All registered	25	Beef	France	France, UK, USA	USA
11	Norwegian Red	21	HapMap^1 ^(1 trio)	21	Dual Purpose	Norway	Norway	Norway
12	Piedmontese	29	24 HapMap^1 ^(3 trios); remaining unrelated	19	Beef	Italy	Italy	Italy
13	Red Angus	15	Registered	15	Beef	Scotland (UK)	USA, Australia	USA
14	Red Poll	23	Registered, a few shared sires and dams	23	Beef	UK		UK
15	Shorthorn	108	Registered (7 trios)	25	Dual Purpose	UK	Global	USA
16	Simmental	777	104 sires; 673 steers from 24 sires	25	Beef	Switzerland	Global	USA
17	Welsh Black	32	several Welsh farms; unrelated	25	Beef	Wales (UK)		UK
	
	Total:	28589		384				

*N*, reference sample size (used to estimate the allele frequencies), ^1 ^HapMap individuals are unrelated except where indicated by 'trio' [45], and, *n*, number of individuals used in assignment testing.

## Results

### Comparison of the marker selection methods

**Figure 1:**
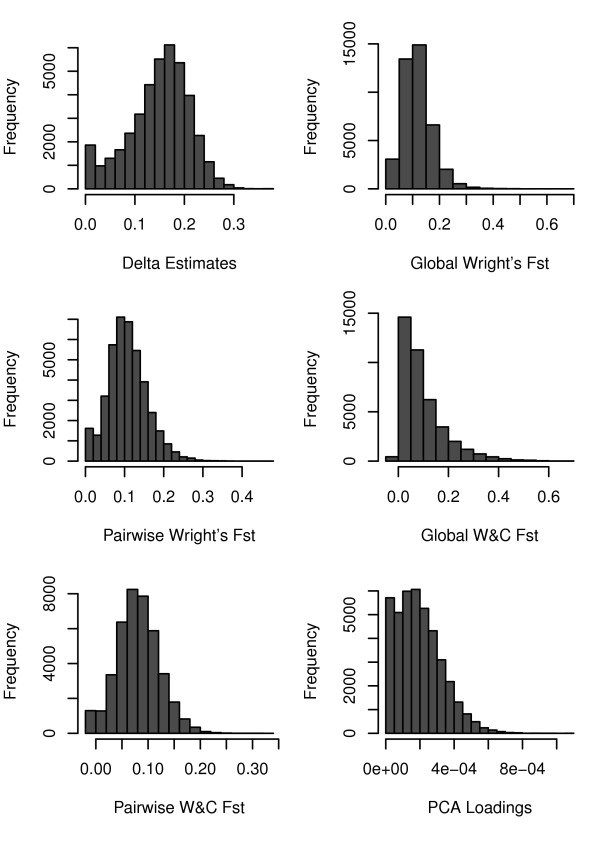
**Frequency histograms of the estimates of genetic information contained in each SNP marker, for each selection method (x-axis scale is method-specific)**. The majority of the SNP markers display low to moderate estimates of genetic informativeness with few markers displaying high levels of population differentiation.

Frequency histograms of the level of genetic information in the SNP markers are shown for each selection method (Figure 1). A predominantly left-skewed distribution was produced for each selection method, except delta, which produced a fairly symmetric distribution. The majority of the markers contained low to medium levels of genetic information and a small proportion had high levels of genetic information (Figure 1).

To assess the level of similarity of the estimates of genetic information contained in each SNP marker across the different selection methods, a Spearman's rank correlation was calculated between the different estimates from the selection methods. High levels of correlation were observed between delta, pairwise Wright's F_ST_, pairwise W&C's F_ST _and PCA (Table 2). Similarly, there was a substantial amount of overlap (> 200) in the top ranked 500 SNP markers between these four selection methods (Table 2). In contrast, the level of correlation was lower between global F_ST _and the other selection methods (Table 2). There was far less overlap (< 200) in the top ranked 500 SNP markers between the global F_ST _estimates and the other selection methods (Table 2).

**Table 2:** Comparison of the SNP selection methods

	delta	**Global Wright's F**_**ST**_	**Pairwise Wright's F**_**ST**_	**Global W&C'S F**_**ST**_	**Pairwise W&C'S F**_**ST**_	PCA [1:8]
**delta**		0.589	0.884	0.370	0.819	0.928
**global Wright's F**_**ST**_	98		0.847	0.462	0.821	0.682
**pairwise Wright's F**_**ST**_	381	151		0.448	0.952	0.888
**global W&C F**_**ST**_	59	49	63		0.461	0.408
**pairwise W&C F**_**ST**_	306	156	367	67		0.810
**PCA [1:8]**	273	101	274	66	229	

The upper-triangle contains the Spearman rank's correlation results between each 40,483 SNPs ranked for information content by each selection method. The lower-triangle contains the amount of overlap for the top 500 ranked SNP markers between each selection method.

To further explore the conflicting results produced by global Wright's and W&C's F_ST_, the observed breed allele frequencies for the top ranked 50 SNP markers for each selection method were displayed in a box-plot [Additional file 1: Supplemental Figure S1]. The boxplot is an effective visual representation of both the central tendency and dispersion of data. Delta, pairwise Wright's F_ST_, pairwise W&C's F_ST _and PCA selected SNP markers with median allele frequency between 0.2 and 0.8 and with large interquartile ranges indicating a high level of dispersion amongst the observed allele frequencies [Additional file 1: Supplemental Figure S1]. In comparison, the majority of the top-ranked SNP markers selected by global Wright's F_ST _had median allele frequencies near 0 or 1 and low levels of dispersion. The global W&C's F_ST _resulted in the selection of SNPs with a higher level of dispersion amongst the observed allele frequencies than global Wright's F_ST_, but, nonetheless, also included markers with quite a few outliers and smaller interquartiles ranges than the other selection methods. The global F_ST _methods resulted in the selection of many SNP markers specific for a single most genetically distinct population.

### Assignment precision: overall assessment

The accuracy of assignment of individual genotypes to known breed origin was evaluated by cumulatively adding 20 markers, in descending order of estimated marker informativeness for each selection method. No population genetic differentiation was detected between the American and British Angus populations (Table 1), consequently the two populations were pooled together and treated as a single breed in subsequent analyses.

**Figure 2:**
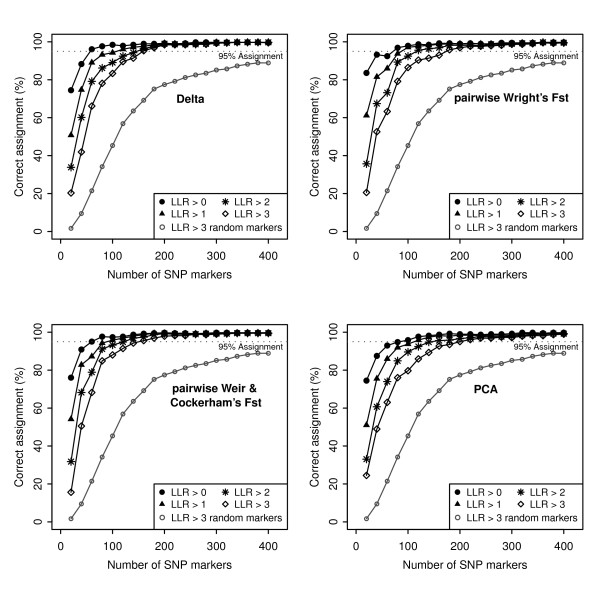
**The percentage assignment success with cumulative number of top-ranked SNP markers at the 4 stringency threshold levels, for each selection method**. 70% success was achieved with the first 20 SNP markers across all ranking methods; power of assignment did not increase beyond 200 SNP markers. Average assignment success across 20 sets of randomly selected markers is also shown for the LLR >3 stringency threshold level.

The success of assignment of the 384 individual genotypes to breed of origin at the four stringency level thresholds for four of the selection methods (delta, pairwise Wright's F_ST_, pairwise W&C's F_ST _and PCA) is presented in Figure 2. Strikingly, it is immediately noticeable that > 50% assignment success for all selection methods is achieved at stringency level LLR > 0 using just the first 20 SNP markers. Overall, pairwise Wright's F_ST _required the smallest number of SNP markers to reach 90%, 95% and 98% correct assignment at the four stringency threshold levels (Table 3). Of the four selection methods, PCA was the poorest performer, requiring > 190 SNP markers to attain 95% assignment success (Figure 2; Table 3). The power of assignment using PCA as a selection method decreased considerably across all the stringency thresholds when a 98% assignment success was imposed (Figure 2; Table 3).

**Table 3:** Individual assignment performance for the four selection methods

	delta			pairwise Wright's F_ST_			pairwise W&C's F_ST_			PCA		
**Log_10_**	**90%**	**95%**	**98%**	**90%**	**95%**	**98%**	**90%**	**95%**	**98%**	**90%**	**95%**	**98%**

0	42.47	59.94	86.48	40.25	57.44	83.72	36.53	62.89	103.07	50.58	75.52	116.07
1	67.97	90.99	129.36	60.12	80.50	114.45	64.37	89.21	129.27	71.85	98.36	152.26
2	95.62	126.63	179.26	80.02	104.62	147.79	89.63	119.13	171.29	101.62	139.54	283.40
3	123.46	159.05	209.70	105.41	137.29	195.69	120.04	159.83	241.62	139.72	192.40	403.89

Estimated number of SNP markers required to achieve 90%, 95% and 98% correct assignment at the four stringency thresholds for each SNP selection method (the individuals from the two Angus populations are pooled). Numbers estimated from asymptotic regression equation.

Full results are not shown for assignment precision using ranked SNP markers for global F_ST _because they performed comparatively poorly. For global Wright's F_ST_, 90% assignment success was obtained with 230 and 380 SNP markers at the stringency levels of LLR > 0 and LLR > 3, respectively. Using up to 400 markers, 95% assignment success was not achieved at any stringency level. For global W&C's F_ST_, 90% assignment success was obtained with 80 and 230 SNP markers at the stringency levels of LLR > 0 and LLR > 3, respectively. The global W&C's F_ST _had greater assignment accuracy over global Wright's F_ST_, but still performed worse than the other four selection methods (Table 3).

Randomly chosen SNP sets performed worse than ranked informative SNP markers in individual assignment analysis (Figure 2). Neither an asymptote nor 95% assignment success were reached using up to 400 markers (average across 20 sets of randomly chosen SNP at LLR > 3).

Individual assignment analysis using a training set and a holdout set was performed in order to evaluate the power of assignment for samples not included in the reference population. This cross-validation analysis reported slightly worse power of assignment than the main analysis [Additional file 1: Supplemental Figure S2]. The assignment power for breeds with large sample sizes N > 50 was comparable to the results of the main analysis (results not shown). However, certain breeds with a low sample size had worse assignment power in the cross-validation analysis. For example, poor assignment power was observed in Red Angus and Norwegian Red, two breeds of low sample size and for which closely related breeds were included in the dataset (Angus and Finnish Ayrshire, respectively) (results not shown).

### Assignment precision: individual breeds

The SNP selection methods differed for power of assignment in individual breeds, but no one method consistently outperformed any other in all breeds (Table 4). No substantial further gain in power of assignment in individual breeds was observed beyond ~ 200 SNP markers. Certain breeds required relatively few SNP markers to attain > 95% assignment success (Table 4). For example, the Jersey breed required < 50 SNPs to achieve 100% individual assignment; even when strict stringency levels were applied. In contrast, the Charolais breed required ~100 SNP markers to achieve > 95% individual assignment and power was severely compromised with increasing stringency level.

**Table 4:** Power of assignment in individual breeds

		Delta				pairwise Wright's F_ST_				pairwise W&C's F_ST_				PCA			
**Breed**	**Markers**	**log0**	**log1**	**log2**	**log3**	**log0**	**log1**	**log2**	**log3**	**log0**	**log1**	**log2**	**log3**	**log0**	**log1**	**log2**	**log3**

Angus	50	100	79.17	66.67	33.33	85.4	64.58	43.75	18.75	93.8	81.25	37.5	16.67	85.4	68.75	52.08	20.83
	100	100	91.67	77.08	72.92	97.9	91.67	89.58	87.5	100	100	95.83	91.67	89.6	79.17	77.08	60.42
	200	100	100	100	100	100	100	100	100	100	100	97.92	97.92	100	97.92	97.92	97.92
	300	100	100	100	100	100	100	100	100	100	100	100	100	100	100	97.92	97.92
	400	100	100	100	100	100	100	100	100	100	100	100	100	100	100	100	100

Brown Swiss	50	100	95.8	95.8	95.8	100	100	100	95.8	100	100	100	91.7	100	100	100	100
	100	100	100	100	100	100	100	100	100	100	100	100	100	100	100	100	100
	200	100	100	100	100	100	100	100	100	100	100	100	100	100	100	100	100
	300	100	100	100	100	100	100	100	100	100	100	100	100	100	100	100	100
	400	100	100	100	100	100	100	100	100	100	100	100	100	100	100	100	100

Charolais	50	72	56	24	0	92	76	60	24	92	60	44	16	88	68	20	4
	100	88	76	56	24	96	96	84	60	96	88	80	44	92	92	84	52
	200	96	96	96	92	96	96	92	92	96	96	96	92	96	96	92	84
	300	100	96	96	96	96	96	96	92	96	96	96	96	96	96	96	96
	400	100	96	96	96	96	96	96	96	96	96	96	96	96	96	96	96

Finnish Ayrshire	50	100	60	20	10	100	90	60	40	70	70	60	50	100	100	70	40
	100	100	100	90	90	100	90	80	50	90	90	80	50	100	100	100	80
	200	100	100	100	100	100	100	100	80	100	100	100	90	100	100	100	100
	300	100	100	100	100	100	100	100	100	100	100	100	100	100	100	100	100
	400	100	100	100	100	100	100	100	100	100	100	100	100	100	100	100	100

Guernsey	50	100	100	95.2	95.2	95.2	95.2	95.2	95.2	100	95.2	95.2	95.2	95.2	95.2	95.2	95.2
	100	100	100	100	95.2	100	100	100	100	100	100	100	100	95.2	95.2	95.2	95.2
	200	100	100	100	100	100	100	100	100	100	100	100	100	100	100	100	100
	300	100	100	100	100	100	100	100	100	100	100	100	100	100	100	100	100
	400	100	100	100	100	100	100	100	100	100	100	100	100	100	100	100	100

Hereford	50	68	60	36	24	92	80	60	48	100	92	84	68	96	88	76	72
	100	100	88	88	84	100	100	96	84	100	100	100	96	100	100	100	100
	200	100	100	100	100	100	100	100	100	100	100	100	100	100	100	100	100
	300	100	100	100	100	100	100	100	100	100	100	100	100	100	100	100	100
	400	100	100	100	100	100	100	100	100	100	100	100	100	100	100	100	100

Holstein	50	96	72	48	24	92	72	64	40	96	96	96	96	96	96	88	84
	100	100	96	96	92	100	100	100	100	100	100	92	88	100	100	92	92
	200	100	100	100	100	100	100	100	100	100	100	100	100	100	100	100	100
	300	100	100	100	100	100	100	100	100	100	100	100	100	100	100	100	100
	400	100	100	100	100	100	100	100	100	100	100	100	100	100	100	100	100

Jersey	50	100	100	100	92.9	100	100	100	100	100	100	100	100	100	100	100	96.4
	100	100	100	100	100	100	100	100	100	100	100	100	100	100	100	100	100
	200	100	100	100	100	100	100	100	100	100	100	100	100	100	100	100	100
	300	100	100	100	100	100	100	100	100	100	100	100	100	100	100	100	100
	400	100	100	100	100	100	100	100	100	100	100	100	100	100	100	100	100

Limousin	50	92	84	56	40	96	92	84	48	88	80	72	44	84	60	20	12
	100	100	100	96	76	100	92	88	84	88	88	72	72	92	92	72	48
	200	100	100	100	100	100	100	100	96	92	92	92	92	100	100	100	100
	300	100	100	96	96	100	96	96	96	96	96	96	92	100	100	100	100
	400	100	100	100	100	100	96	96	96	100	96	96	96	100	100	100	100

Norwegian Red	50	90.5	71.4	61.9	33.3	90.5	71.4	57.1	28.6	90.5	81	71.4	57.1	85.7	76.2	61.9	28.6
	100	100	95.2	90.5	85.7	95.2	90.5	85.7	76.2	90.5	90.5	76.2	71.4	95.2	95.2	90.5	85.7
	200	100	100	100	100	100	100	100	95.2	100	100	100	95.2	95.2	100	100	95.2
	300	100	100	100	100	100	100	100	100	100	100	100	100	100	100	100	100
	400	100	100	100	100	100	100	100	100	100	100	100	100	100	100	100	100

Piedmontese	50	100	94.7	94.7	78.9	100	100	100	94.7	100	94.7	94.7	73.7	94.7	84.2	73.7	47.4
	100	100	100	100	94.7	100	100	100	100	100	100	100	100	94.7	94.7	94.7	68.4
	200	100	100	100	100	100	100	100	100	100	100	100	100	100	100	100	100
	300	100	100	100	100	100	100	100	100	100	100	100	100	100	100	100	100
	400	100	100	100	100	100	100	100	100	100	100	100	100	100	100	100	100

Red Angus	50	93	73.3	46.7	26.7	86.7	53.3	33.3	20	80	53.3	46.7	13.3	93.3	53.3	46.7	20
	100	93	80	66.7	60	86.7	86.7	80	66.7	100	93.3	93.3	73.3	93.3	80	73.3	60
	200	93	93.3	93.3	93.3	100	100	100	93.3	100	100	93.3	93.3	100	93.3	80	80
	300	100	100	100	100	100	100	100	100	100	100	100	100	100	86.7	86.7	86.7
	400	100	100	100	100	100	100	100	100	100	100	100	100	100	93.3	93.3	93.3

Red Poll	50	88.9	88.9	83.3	72.2	100	100	83.3	77.8	94.4	88.9	77.8	66.7	100	94.4	94.4	94.4
	100	100	100	100	100	100	100	100	94.4	100	100	100	100	100	100	100	100
	200	100	100	100	100	100	100	100	100	100	100	100	100	100	100	100	100
	300	100	100	100	100	100	100	100	100	100	100	100	100	100	100	100	100
	400	100	100	100	100	100	100	100	100	100	100	100	100	100	100	100	100

Shorthorn	50	80	76	68	56	92	92	92	80	92	92	92	88	96	92	80	80
	100	92	88	88	88	92	92	92	88	96	96	92	92	100	96	96	92
	200	96	96	92	92	100	96	96	96	100	96	96	96	100	100	100	100
	300	96	100	100	100	100	96	96	96	100	96	96	96	100	100	100	100
	400	100	100	100	96	100	100	100	100	100	100	100	100	100	100	100	100

Simmental	50	100	92	68	36	92	92	80	60	96	84	68	40	88	68	44	32
	100	100	92	84	80	96	100	96	96	100	100	88	76	92	92	76	56
	200	100	100	100	96	100	100	100	100	100	100	96	92	92	88	76	68
	300	100	100	100	96	100	100	100	100	100	96	96	96	96	92	88	76
	400	100	100	100	100	100	100	100	100	100	100	100	100	100	100	96	92

Welsh Black	50	100	100	93.3	76.7	96.7	93.3	83.3	80	100	96.7	90	83.3	96.7	96.7	90	83.3
	100	100	100	100	100	96.7	93.3	93.3	93.3	100	100	100	100	96.7	96.7	96.7	96.7
	200	100	100	100	100	100	100	100	100	100	100	100	96.7	100	100	96.7	96.7
	300	100	100	100	100	100	100	100	100	100	100	100	100	100	96.7	96.7	96.7
	400	100	100	100	100	100	100	100	100	100	100	100	100	100	100	100	100

Percentage of individuals that were successfully assigned to their breed origin, at the 4 stringency threshold levels, for each selection method.

**Figure 3:**
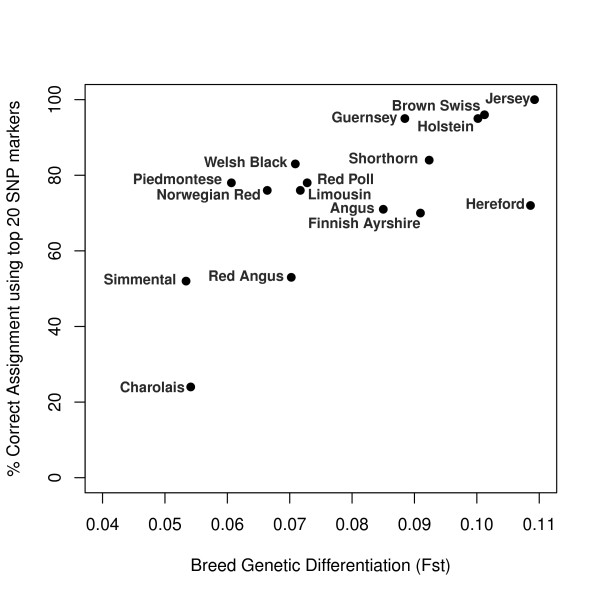
Scatterplot of average pairwise breed genetic differentiation correlated against percentage correct assignment using the top-ranked 20 SNP markers (Wright's Fst method; Spearman's rank correlation, r = 0.635).

There was a positive significant correlation between the percentage of correctly assigned individuals and a breed's average level of genetic differentiation (Figure 3; Spearman's rank correlation, rho = 0.635, p = 0. 0082).

Type I (false positives) and II errors (false negatives) that occurred in the individual assignment analysis, using pairwise Wright's F_ST _at the lowest stringency threshold level (LLR > 0) were calculated [Additional file 1: Supplemental Table S1]. Using 50 SNP markers, 5 breeds were assigned with 100% assignment success, and the remaining breeds had type I errors of < 15%. The type I error rate was highest for Angus (14.6%), followed closely by Red Angus (13.3%), whereby if an individual was not assigned to its correct origin it was assigned to the other breed. Using 50 SNP markers, eight breeds had no individuals assigned from other breeds, and the remaining breeds displayed a type II error of < 17% (except for the Red Angus breed, where 35% of the assigned individuals were Angus; and this may have been inflated by the relatively low sample size of Red Angus breed (15), compared to Angus (41)). The type I and II error rates decreased to < 5% by 150 SNP markers.

### Ascertainment bias

The SNP markers on the BovineSNP50 BeadChip were discovered through various breed sources. The majority of the markers were discovered from Angus, Holstein and Hereford breeds (others included Charolais, Limousin, Red Angus, Simmental, Jersey, Limousin and Norwegian Red, but fewer SNPs were found through these breeds) [18]. The inclusion of few representative sources could influence the level of SNP informativeness and individual assignment power, such that breeds used in the discovery process show higher SNP variability. Although Jersey was one of the breeds used for SNP discovery, it had the lowest average minor allele frequency (MAF) (Table 5). MAF values for Angus, Hereford and Holstein were relatively high but lower than for Charolais and Simmental. The power of assignment at a breed level revealed that the breeds represented during the SNP discovery process were not amongst those (except for Jersey) that required comparatively fewer markers to achieve 100% assignment success (Table 4).

**Table 5:** Average minor allele frequency for each breed across the 40, 483 SNP markers

Breed	MAF
Angus	0.230
Brown Swiss	0.199
Charolais	0.243
Finnish Ayrshire	0.219
Guernsey	0.202
Hereford	0.236
Holstein	0.231
Jersey	0.196
Limousin	0.230
Norwegian Red	0.227
Piedmontese	0.230
Red Angus	0.218
Red Poll	0.215
Shorthorn	0.204
Simmental	0.244
Welsh Black	0.221

The top 500 SNP markers ranked by decreasing informativeness were listed with their corresponding SNP discovery method (7 in total, [18]) [Additional file 2: Supplemental Table S2]. A *x*^2^-test revealed that the proportions of SNP discovery methods represented in the pairwise Wright's F_ST _500 top SNP markers [Additional file 2: Supplemental Table S2] were not significantly different from those of the overall Bovine SNP50 set (*x*^2^, df = 36, NS).

## Discussion

The principal goal of this study was to evaluate marker selection methods and determine the minimum number of SNP markers from the BovineSNP50 BeadChip required to effectively and confidently assign individual genotypes to European cattle breeds. While all SNP selection methods yielded reduced marker panels capable of breed identification, the power of assignment varied markedly among analysis methods.

### Behaviour of the marker selection methods

The pairwise Wright's F_ST _selection method marginally outperformed other selection methods in the individual assignment analysis (Table 3, Figure 2). Nonetheless, three other selection methods, delta, pairwise W&C's F_ST _and PCA, did not perform poorly at ranking markers or for assignment success rates. Across these selection methods, to achieve 95% assignment success, < 80, < 100, < 140 and < 200 SNP markers were required at the stringency threshold levels of LLR > 0, LLR > 1, LLR > 2 and LLR > 3, respectively (Table 3, Figure 2). These four selection methods (delta, pairwise Wright's F_ST_, pairwise W&C's F_ST _and PCA) to a large extent agreed on the most informative SNP markers. The resulting estimates of genetic informativeness of each SNP marker were highly correlated across the four selection method and there was a large degree of overlap among the top-ranked 500 SNP markers (Table 2). This was to be expected because all methods were applied to individual SNP marker allele frequencies. In addition, it has been demonstrated that delta and Wright's F_ST _function similarly [31]. However, PCA exhibited the poorest correlation with the other methods and lowest overall individual assignment power. Paschou *et al.*, [24] advocated using PCA to determine marker informativeness because PCA renders an overall estimate for a SNP marker, as compared with other selection methods where it is necessary to estimate an average from pairwise calculations when the number of populations (K) > 2. PCA is an approach used to characterise the structure of a set of variables (in this case SNPs). The inferred relationships between objects (e.g., populations/breeds) are determined by the structure of the covariance matrix between the marker allele frequencies. Thus, the informativeness of a given marker will depend on the other markers included in the analysis and this could influence the informative markers that PCA identified. In contrast, delta and F_ST _do not take into account the relationships amongst markers and the level of information of each marker is estimated independently of the others.

The remaining two selection methods, global Wright's and W&C's F_ST_, performed comparatively poorly in the individual assignment test. As similarly observed by Kersbergen *et al. *[32], global F_ST _may not be appropriate to assess the level of genetic information in SNP markers when K > 2, as the method could result in the selection of SNP markers which are specific in distinct populations [Additional file 1: Supplemental Figure S1]. The selected SNP markers that were specific for only the most distinct breed were not segregating in the majority of the other breeds [Additional file 1: Supplemental Figure S1], and thus the expected heterozygosity would be low. Indeed, it is suggested that genetic markers with high expected heterozygosity are informative and therefore useful in individual assignment analysis [15,33], such as those identified using pairwise Wright's F_ST_, delta, pairwise W&C's F_ST _and PCA. As a result the performance of individual assignment tests using global F_ST _selected markers may be compromised compared to the other selection methods. Consequently, when K > 2 it is preferable to estimate F_ST_, either Wright's or W&C's, on a population pairwise basis and then estimate the average across the pairwise comparisons to obtain an overall estimate for a marker.

### Assignment precision: minimum number of markers required

Since pairwise Wright's F_ST _outperformed the other selection methods (Table 3) this selection method was subsequently adopted to estimate the minimum number of SNP markers required to achieve the desired assignment success. At the most commonly used stringency threshold (LLR > 0) and the accepted level of appropriate assignment success (95%) [34], < 60 SNP markers were required for the correct assignment of the 384 individual genotypes. When stricter stringency threshold levels are applied, the number of SNP markers required to attain 95% assignment success increased (Table 3). Depending on the chosen degree of confidence, the required number of markers ranges from 60 to 140 SNPs (80, 105 and 140 at LLR > 1, LLR > 2 and LLR > 3, respectively). While the percentage of assignment success decreases with increasing stringency thresholds, so too does the risk of false assignment. Consequently, there is greater confidence in the estimated genotype likelihoods and LLR calculations if a strict stringency threshold (LLR > 3) is adopted.

It is difficult to compare the results obtained here to other studies conducted on individual assignment analysis in cattle breeds. First, most previous studies used microsatellite markers and, second, these studies had only a limited number of markers (e.g., [5,8]). These studies also primarily focused on the practicality of assigning individuals among cattle breeds with the available markers and were not concerned with how many markers would be required to achieve confident assignment of individual genotypes. In a study of French cattle breeds, Maudet *et al.*, [8] found that using 23 microsatellite loci > 93% of individuals could be assigned to their breed origin. A more recent study used SNP markers but did not have a large dataset at their disposal and could, again, only address the practicality of individual assignment with the limited set of available markers [9]. Using 90 SNP markers genotyped in 24 European cattle breeds they were able to correctly assign 85% of individuals to their breed origin. McKay *et al.*, [35] used STRUCTURE to assess the number of loci required to estimate the number of ancestral populations in 6 *Bos taurus *breeds. The use of 150 randomly chosen loci (from a dataset of 2,641 loci) yielded the correct number of clusters in only 40% of cases, consistent with reduced assignment power for randomly-selected markers found in the current study (Figure 2). The lower assignment power in those studies was most probably a direct consequence of using an insufficient number of informative loci. The comparatively high assignment power of fewer SNP markers in the current study was probably due to the availability of > 40,000 SNP markers and the benefit of selecting markers that contain the most genetic information with respect to the reference populations. Only a few highly polymorphic microsatellite loci are required in individual assignment studies. However, dense SNP panels are now available for many species and SNP markers possess numerous advantages, including cost, throughput and reliability, making them a favourable choice over microsatellites.

### Assignment success: individual breeds

It is evident that certain breeds in this study require far fewer markers to achieve > 95% assignment success than others, regardless of the selection method used (Table 4, Figure 3). For example, the Jersey, Brown Swiss, Guernsey and Piedmontese breeds achieved 100% assignment success, even at stricter stringency thresholds using 50 SNP markers (pairwise Wright's F_ST_, LLR > 2, Table 4). In contrast, the French breeds like the Charolais, Limousin and Simmental achieved ~ 90% assignment success at LLR > 0, which fell to < 50% with increasing stringency threshold using 50 SNP markers (Table 4). Similarly, the breeds that exhibited a lower power of assignment success (Table 4) also had higher type I and II error rates (Table S1).

A problem associated with the use of SNP markers in population genetics is ascertainment bias, which could influence population genetic estimates and may contribute to differences in assignment performance for individual breeds [10]. Heterogeneity amongst sample representatives can introduce ascertainment bias and breeds not included in the SNP discovery process could have lower minor allele frequencies (MAF) [15,36]. The average MAF was lowest in the Brown Swiss, Guernsey and Jersey breeds (Table 5), one of which was represented in the SNP discovery process and the three breeds which were central to the process (Angus, Hereford, Holstein) did not have the highest average MAF values. In addition, no one particular SNP discovery method was over-represented in the top identified SNP markers [Additional file 2: Supplemental Table S2] as the discovery method proportions were similar to that represented on the Bovine SNP50 assay [18]. SNP ascertainment bias would have been more pronounced if *B. t. indicus *breeds had been included in this study [36]. Morin *et al.*, [10] concluded that ascertainment bias may be an issue in the assessment of population size and demographic changes. It is least important for individual identification and assignment tests, where the intentional selection of informative markers provides greater power than do randomly chosen markers.

A factor that could affect the power of assignment success and variation in power of assignment between breeds is the level of pairwise genetic differentiation amongst the breeds. It is known that the number of markers required to obtain a high accuracy of assignment is influenced by the level of population genetic differentiation [8,37]. That is, it depends closely on the populations under consideration and respective levels of genetic heterogeneity. As demonstrated in Figure 3, the level of genetic differentiation of a breed, measured by F_ST_, is correlated with power of assignment success. Low breed genetic differentiation was observed in Charolais and Simmental, which similarly showed higher rates of Type I and II errors (Figure 3, [Additional file 1: Supplemental Table S1]). False positive assignments also occurred between breeds of known recent ancestry, for example, Angus and Red Angus, and Finnish Ayrshire and Norwegian Red [36]. In addition, cases of mistaken assignment occurred between Charolais, Simmental, Limousin and Shorthorn, where the pairwise F_ST _values amongst these breeds were < 0.1. In a study on individual assignment using microsatellites, Ciampolini *et al.*, [5] reported that of the four breeds under consideration, Charolais and Limousin had the lowest level of pairwise genetic differentiation and were the most difficult to discriminate between (F_ST _= 0.041). As assignment success is a function of both the number of markers and population genetic differentiation, the level of breed genetic differentiation is indicative of the potential number of SNP markers necessary to attain high levels of power in individual assignment tests [6,37].

### Informative marker panels in population genetics

Evaluation of the selection methods revealed that only a small proportion of the markers from the BovineSNP50 BeadChip were highly informative for discriminating among 17 breeds, and the majority contained medium to low levels of genetic information (Figure 1). This is consistent with the development of the assay in which SNPs with high MAF across *B. t. taurus *breeds were preferentially selected in the assay design. Consequently, sets of randomly chosen SNP markers contained sufficient genetic information to produce moderate levels of individual assignment power (Figure 2). However, in contrast, a substantially reduced set of highly informative SNP markers were capable of precisely discriminating amongst the European cattle breeds (Figure 2).

Studies have shown that a reduced set of selected informative markers can effectively capture the genetic structure of human populations [23,24]. For instance, Lao *et al.*, [23] found that 10 SNP markers from a 10K SNP array contained enough genetic information to differentiate individuals from Africa, Europe, Asia and America and additional loci contributed very little extra information. Indeed, it is generally considered that uninformative markers (i.e., monomorphic loci) may add noise to the results and compromise power of population genetic studies [38,39]. It could be useful to create a minimum panel of maximum power, particularly when using Bayesian genotypic clustering software such as STRUCTURE to elucidate population structure, because these approaches are computationally demanding (which intensifies as the number of markers increases) [23]. Consequently, it is practical and cost-effective to apply a selection method to dense assays to isolate the highly diagnostic markers and increase the power of analysis.

The number of markers required for population assignment will depend on the species, the populations under consideration, their respective level of genetic differentiation and the desired stringency of assignment. For instance, within dogs 27% of the genetic variation is found between breeds, whereas for humans the level between populations is only 5%-10% [40]. As a result, the number of SNP markers required for individual assignment and discrimination amongst populations (breeds) will differ between species under consideration.

## Conclusion

Although the marker selection methods explored in this study agreed to a large extent on which SNPs were the most informative, there were significant differences in the power of assignment produced by the resulting ranked SNP panels, with pairwise Wright's F_ST _outperforming all other approaches. These results illustrate that with effective exploration it is possible to identify the most informative markers and produce an optimal minimum set of markers that can differentiate among populations.

## Methods

### Data

Allele frequencies from 17 cattle breeds representing the 'reference' populations and a total of 384 individual genotypes of known breed origin, sampled from the reference populations, were available (Table 1). Information on the sampling of the reference populations is given in Table 1. Decker *et al.*, [36] selected 40,843 SNPs from the Bovine SNP50 Bead Chip after a strict quality screening where "Loci selected for analysis were all located on autosomes, had a call rate of at least 80% in 36 (75%) *B. t. taurus *breeds, and were not monomorphic in all breeds.... ". Since only *B. t. taurus *breeds were used in the current study the selected set of SNP markers by Decker *et al.*, [36] was adopted. Detailed information of the genotyping procedure can be found in Decker *et al.*, [36].

### Selection methods to determine the most informative markers

The breed-specific allele frequencies for the 40,483 SNPs were used to estimate the genetic information contained in each SNP marker using the following selection methods: delta, Wright's F_ST_, Weir and Cockerham's F_ST _and PCA. The larger the estimated value, the more informative the marker is at genetically discriminating the sampled populations. All analyses were conducted in the R statistical environment [41].

### Delta

One of the most commonly used measures of marker informativeness is delta [25]. For a biallelic marker the delta value is given by | p_A*i *_- p_A*j *_|, where p_A*i *_and p_A*j *_are the frequencies of allele A in the *i*^th ^and *j*^th ^populations, respectively. Delta can only be estimated between pairs of populations (K = 2). Since K = 17 in this study, values were averaged across all pairwise comparisons to produce an estimated value for each SNP marker.

### F_ST_

Wright [28] introduced F-statistics to describe the proportion of genetic diversity within and among populations [42]. Wright's F_ST _statistic has been extended by several authors and a preferable statistic based on the analysis of variance of allele frequencies is Weir and Cockerham's (W&C) F_ST _[29]. For both methods unbiased estimates of F_ST _were first calculated over all populations (global F_ST_) and on a pairwise basis (pairwise F_ST_), with the latter values being averaged over all pairs to produce an estimated information content value for each SNP marker.

#### Wright's F_ST_

Wright's F_ST _was estimated as , where var(*p*_*A*_) is the variance of the allele frequency among breeds and  is the mean allele frequency across the breeds.

#### W&C's F_ST_

Unbiased estimates of W&C's F_ST _were estimated as functions of variance components as detailed in Akey *et al.*, [43]. Estimated F_ST _can be negative if alleles drawn at random from within a population are less similar to one another than those drawn from different populations (F_ST _< 0) [43,44]. In this study the estimated F_ST _values were left as negative.

### Principal Component Analysis (PCA)

PCA is a statistical technique that can be used to reduce the dimension of a multivariate dataset. The original variables are linearly transformed by PCA into a set of underlying variables ("principal components") ranked in terms of their variance, such that most of the original variability may be contained in a smaller number of variables. Each new variable has an associated eigenvalue that measures the respective amount of explained variance. The coefficients ("loadings") used in the linear transformation of the original variables into new variables generate the proportion of variance that a variable contributes to a given principal component.

PCA was performed following Paschou *et al.*, [24], but on the breed-specific allele frequency matrix rather than the individual genotypes. To determine which principal components were significant, 100 random matrices were created by sampling with replacement allele frequencies within each SNP marker across all breeds. The first eight principal components for the actual data contained more information than in the randomly generated components (i.e., their eigenvalues were greater) and therefore the first eight principal components were used to calculate marker informativeness. The loadings for each SNP marker were squared and summed over the eight significant principal components to produce an estimate of informativeness [24].

### Individual Assignment Analysis

Several genetic assignment approaches are available [6,7,37]. The Bayesian implementation developed by Rannala and Mountain [6] has been found to be more effective at individual assignment than other methods [37]. However, the method of Paetkau *et al.*, [7] is equally effective at individual assignment when the levels of genetic differentiation between reference populations are high [37]. Comparison of the two methods for a subset of cattle breeds from the current study revealed similar performance levels (results not shown). Consequently, the method of Paetkau *et al.*, [7] was employed as it is easier to implement than that of Rannala & Mountain [6] and is most frequently employed in empirical studies.

Allele frequencies of zero were replaced by a value of 1 × 10^-5 ^because log(0) is not defined [7]. Likewise, if an observed allele frequency was 1, it was replaced by a value of 0.99999.

Genotype likelihoods were calculated for each individual in each reference population based on the observed allele frequencies for each marker. Let *p*_*ijk *_denote the frequency of the *k*^th ^allele (*k *= 1, 2) at the *j*^th ^locus (*j *= 1 .. *J*) in the *i*^th ^population (*I *= 1 .. *I*). Let *g*_*jkk' *_denote an individual's diploid genotype at the *j*^th ^locus, and let the Mendelian transmission probability of *g*_*jkk' *_arising in the *i*^th ^population be *T*(*g*_*jkk' *_| *i*)

where a genotype is homozygous if *k *= *k*' and heterozygous otherwise, under the assumption of random union of gametes. Next, let *g *denote an individual's multilocus genotype. The likelihood of an individual diploid genotype occurring in a particular population, *T*(*g*|*i*), was estimated as above, as the square of the observed allele frequency for homozygotes or twice the product of the two allele frequencies for heterozygotes. Under the assumption of independence between the *J *loci

and

To assess the performance of the assignment procedure, log-likelihood ratios (LLR) were calculated by comparing the likelihood of an individual being assigned to its population of origin and the likelihood of it being assigned to another population

Different stringency thresholds can be applied as confidence levels of assignment precision. Four stringency levels are commonly used: LLR > 0, LLR > 1, LLR > 2 and LLR > 3 [4,25,26,34]. LLR > 1, LLR > 2 and LLR > 3 levels, respectively, mean that a multilocus genotype has to be 10, 100 or 1000 times more likely in one population than any other. The LLR > 0 level requires that the genotype to be more likely in one population than any other. The correct assignment of an individual genotype to its known origin occurred when the calculated LLR was greater than the selected stringency level. If the LLR was lower than the selected stringency level, the individual genotype failed to be assigned to its origin and was instead assigned to the reference population that yielded the highest overall log-likelihood.

To obtain an estimate of the number of SNP markers required to achieve 90%, 95% and 98% correct assignment success of the 384 individual genotypes for each of the selection methods, at each of the 4 threshold levels, a non-linear regression model was fitted to the curves of correct assignment percentage against cumulative markers. An asymptotic regression model (*y *= *a *+ *b *exp^*cx*^, where parameter *a *represents the value of the asymptote, parameter *b *represents the difference between the value of *y *when *x *= 0 and the upper asymptote and parameter *c *represents the natural logarithm of the rate of exponential increase) was found to best fit the data. When *a *> 0, *b *< 0 and *c *< 0 the model represents the law of diminishing returns in which the rate of increase of *y *declines with successive equal increments of *x*.

To test whether the level of genetic differentiation of a breed corresponded to the power of assignment, a Spearman's rank correlation was calculated between the percentage of correctly assigned individuals for the 20 top ranked SNP markers for each breed (selection method = pairwise Wright's F_ST_, LLR > 0) and the average F_ST _for each breed (pairwise Wright's F_ST _values across all breeds, based on 40, 843 SNP markers, averaged to provide an estimate for each breed).

A negative control to individual assignment analysis was applied by analysing 20 sets of 400 randomly selected SNPs. The average individual assignment success was estimated across the 20 random SNP sets at the stringency level LLR > 3.

In order to evaluate the power of assignment for samples of unknown origin, the individual assignment analysis was evaluated by cross-validation whereby a training sample was used to identify the informative loci and a holdout sample from each of the breeds was used to test the power of the resulting panel and the reference training sample. For breeds with a reference sample size > 50 (Table 1) the holdout sample comprised all the individuals to be assigned (those in column *n*); these were removed from their respective reference breed and allele frequencies of the reference breeds were re-estimated. For breeds with a reference sample size < 50 (Table 1) five random individual genotypes of the individuals assigned in the main analysis (those in column *n*) were designated as the holdout sample; these were removed from their respective reference breed and allele frequencies were re-estimated. The individual assignment analysis was repeated with the new training samples and the hold-out samples.

## Authors' contributions

SW participated in the study design, wrote the computer code, carried out the statistical analysis and drafted the manuscript. PW participated in the study design and manuscript preparation. ALA was a co-PI, involved in project design and manuscript preparation. AL provided bioinformatics support. RDS called the genotypes in BeadStudio, performed QA/QC analyses and estimated allele frequencies.

SDM genotyped all of the samples. JFT collected the samples and DNAs were extracted and genotyped in his laboratory. RO was the Principal Investigator on the project responsible for its conception, funding and implementation. All authors contributed to the writing and read and approved the final version of the manuscript.

## Supplementary Material

Additional file 1 **Supplemental materials**. Figure S1: A boxplot of the observed breed allele frequencies for the top ranked 50 SNP markers for each selection method. Figure S2: A plot of the percentage assignment success with cumulative number of top-ranked SNP markers at the 4 stringency threshold levels. The results of this individual assignment test is for the training set and hold-out set where the selection implemented was Wright's pairwise F_ST_. Table S1: Type I (false positives) and II errors (false negatives). The table details the error rates that occurred in the individual assignment analysis, using pairwise Wright's F_ST _at the lowest stringency threshold level (LLR > 0). Click here for file

Additional file 2 **Table S2**. Top 500 SNP markers. The genetic markers are ranked by decreasing informativeness and the corresponding SNP discovery methods are listed with each SNP marker. Click here for file
