# Supplementary material for: Evaluation of approaches for identifying population informative markers from high density SNP Chips
Source: BMC Genet. 2011 May 13;12:45. doi: 10.1186/1471-2156-12-45 (PMC3118130; doi:10.1186/1471-2156-12-45)
Supplement: Additional file 1 — Supplemental materials. Figure S1: A boxplot of the observed breed allele frequencies for the top ranked 50 SNP markers for each selection method. Figure S2: A plot of the percentage assignment success with cumulative number of top-ranked SNP markers at the 4 stringency threshold levels. The results of this individual assignment test is for the training set and hold-out set where the selection implemented was Wright's pairwise FST. Table S1: Type I (false positives) and II errors (false negatives). The table details the error rates that occurred in the individual assignment analysis, using pairwise Wright's FST at the lowest stringency threshold level (LLR > 0). [file 1471-2156-12-45-S1.DOC]

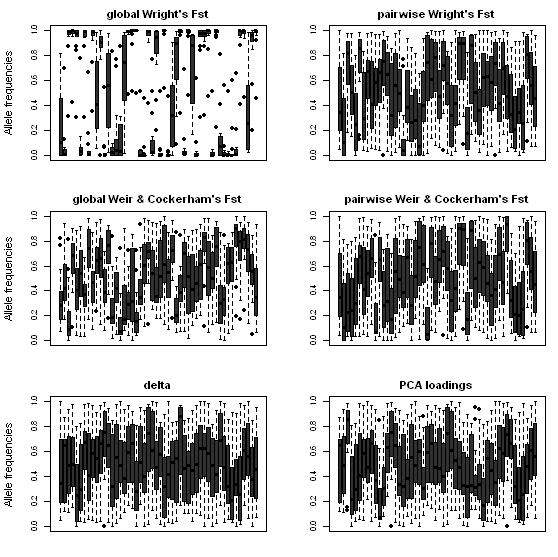


Figure S1. Boxplot of the observed allele frequencies in each reference population for the 50 top-ranked SNP markers from each selection method.

| TYPE I ERROR | | BREED OF ORIGIN | | | |  |  |  |  |  |  |  |  |  |  |  |  |
| --- | --- | --- | --- | --- | --- | --- | --- | --- | --- | --- | --- | --- | --- | --- | --- | --- | --- |
| ALLOCATED  BREED |  | A | BS | CH | FA | GU | HR | HO | JR | LIM | NR | PD | RA | RP | SH | SIM | WB |
| 50 SNPs | Angus | 85 | 0 | 0 | 0 | 0 | 0 | 0 | 0 | 0 | 0 | 0 | 13.3 | 0 | 0 | 0 | 0 |
|  | Brown Swiss | 0 | 100 | 0 | 0 | 0 | 0 | 0 | 0 | 0 | 0 | 0 | 0 | 0 | 0 | 0 | 0 |
|  | Charolais | 0 | 0 | 92 | 0 | 0 | 4 | 8 | 0 | 0 | 0 | 0 | 0 | 0 | 4 | 0 | 0 |
|  | Finnish Ayrshire | 0 | 0 | 0 | 100 | 0 | 0 | 0 | 0 | 0 | 9.5 | 0 | 0 | 0 | 0 | 0 | 0 |
|  | Guernsey | 0 | 0 | 0 | 0 | 95.2 | 0 | 0 | 0 | 0 | 0 | 0 | 0 | 0 | 0 | 0 | 0 |
|  | Hereford | 0 | 0 | 0 | 0 | 0 | 92 | 0 | 0 | 0 | 0 | 0 | 0 | 0 | 0 | 0 | 0 |
|  | Holstein | 0 | 0 | 0 | 0 | 0 | 0 | 92 | 0 | 0 | 0 | 0 | 0 | 0 | 0 | 0 | 0 |
|  | Jersey | 0 | 0 | 0 | 0 | 0 | 0 | 0 | 100 | 0 | 0 | 0 | 0 | 0 | 0 | 0 | 0 |
|  | Limousin | 0 | 0 | 0 | 0 | 0 | 0 | 0 | 0 | 96 | 0 | 0 | 0 | 0 | 0 | 4 | 0 |
|  | Norwegian Red | 0 | 0 | 0 | 0 | 0 | 0 | 0 | 0 | 0 | 90.5 | 0 | 0 | 0 | 0 | 0 | 0 |
|  | Piedmontese | 0 | 0 | 0 | 0 | 4.8 | 0 | 0 | 0 | 4 | 0 | 100 | 0 | 0 | 0 | 4 | 3.3 |
|  | Red Angus | 15 | 0 | 0 | 0 | 0 | 0 | 0 | 0 | 0 | 0 | 0 | 86.7 | 0 | 0 | 0 | 0 |
|  | Red Poll | 0 | 0 | 0 | 0 | 0 | 4 | 0 | 0 | 0 | 0 | 0 | 0 | 100 | 0 | 0 | 0 |
|  | Shorthorn | 0 | 0 | 0 | 0 | 0 | 0 | 0 | 0 | 0 | 0 | 0 | 0 | 0 | 92 | 0 | 0 |
|  | Simmental | 0 | 0 | 8 | 0 | 0 | 0 | 0 | 0 | 0 | 0 | 0 | 0 | 0 | 4 | 92 | 0 |
|  | Welsh Black | 0 | 0 | 0 | 0 | 0 | 0 | 0 | 0 | 0 | 0 | 0 | 0 | 0 | 0 | 0 | 96.7 |
| 100 SNPs | Angus | 98 | 0 | 0 | 0 | 0 | 0 | 0 | 0 | 0 | 0 | 0 | 13.3 | 0 | 0 | 0 | 0 |
|  | Brown Swiss | 0 | 100 | 0 | 0 | 0 | 0 | 0 | 0 | 0 | 0 | 0 | 0 | 0 | 0 | 0 | 0 |
|  | Charolais | 0 | 0 | 96 | 0 | 0 | 0 | 0 | 0 | 0 | 0 | 0 | 0 | 0 | 4 | 0 | 3.3 |
|  | Finnish Ayrshire | 0 | 0 | 0 | 100 | 0 | 0 | 0 | 0 | 0 | 4.8 | 0 | 0 | 0 | 0 | 0 | 0 |
|  | Guernsey | 0 | 0 | 0 | 0 | 100 | 0 | 0 | 0 | 0 | 0 | 0 | 0 | 0 | 0 | 0 | 0 |
|  | Hereford | 0 | 0 | 0 | 0 | 0 | 100 | 0 | 0 | 0 | 0 | 0 | 0 | 0 | 0 | 0 | 0 |
|  | Holstein | 0 | 0 | 0 | 0 | 0 | 0 | 100 | 0 | 0 | 0 | 0 | 0 | 0 | 0 | 0 | 0 |
|  | Jersey | 0 | 0 | 0 | 0 | 0 | 0 | 0 | 100 | 0 | 0 | 0 | 0 | 0 | 0 | 0 | 0 |
|  | Limousin | 0 | 0 | 0 | 0 | 0 | 0 | 0 | 0 | 96 | 0 | 0 | 0 | 0 | 0 | 0 | 0 |
|  | Norwegian Red | 0 | 0 | 0 | 10 | 0 | 0 | 0 | 0 | 0 | 95.2 | 0 | 0 | 0 | 0 | 0 | 0 |
|  | Piedmontese | 0 | 0 | 0 | 0 | 0 | 0 | 0 | 0 | 0 | 0 | 100 | 0 | 0 | 0 | 0 | 0 |
|  | Red Angus | 2 | 0 | 0 | 0 | 0 | 0 | 0 | 0 | 0 | 0 | 0 | 86.7 | 0 | 0 | 0 | 0 |
|  | Red Poll | 0 | 0 | 0 | 0 | 0 | 0 | 0 | 0 | 0 | 0 | 0 | 0 | 100 | 0 | 0 | 0 |
|  | Shorthorn | 0 | 0 | 0 | 0 | 0 | 0 | 0 | 0 | 0 | 0 | 0 | 0 | 0 | 92 | 0 | 0 |
|  | Simmental | 0 | 0 | 4 | 0 | 0 | 0 | 0 | 0 | 4 | 0 | 0 | 0 | 0 | 4 | 100 | 0 |
|  | Welsh Black | 0 | 0 | 0 | 0 | 0 | 0 | 0 | 0 | 0 | 0 | 0 | 0 | 0 | 0 | 0 | 96.7 |
| 150 SNPs | Angus | 100 | 0 | 0 | 0 | 0 | 0 | 0 | 0 | 0 | 0 | 0 | 6.7 | 0 | 0 | 0 | 0 |
|  | Brown Swiss | 0 | 100 | 0 | 0 | 0 | 0 | 0 | 0 | 0 | 0 | 0 | 0 | 0 | 0 | 0 | 0 |
|  | Charolais | 0 | 0 | 96 | 0 | 0 | 0 | 0 | 0 | 0 | 0 | 0 | 0 | 0 | 4 | 0 | 0 |
|  | Finnish Ayrshire | 0 | 0 | 0 | 100 | 0 | 0 | 0 | 0 | 0 | 0 | 0 | 0 | 0 | 0 | 0 | 0 |
|  | Guernsey | 0 | 0 | 0 | 0 | 100 | 0 | 0 | 0 | 0 | 0 | 0 | 0 | 0 | 0 | 0 | 0 |
|  | Hereford | 0 | 0 | 0 | 0 | 0 | 100 | 0 | 0 | 0 | 0 | 0 | 0 | 0 | 0 | 0 | 0 |
|  | Holstein | 0 | 0 | 0 | 0 | 0 | 0 | 100 | 0 | 0 | 0 | 0 | 0 | 0 | 0 | 0 | 0 |
|  | Jersey | 0 | 0 | 0 | 0 | 0 | 0 | 0 | 100 | 0 | 0 | 0 | 0 | 0 | 0 | 0 | 0 |
|  | Limousin | 0 | 0 | 0 | 0 | 0 | 0 | 0 | 0 | 100 | 0 | 0 | 0 | 0 | 0 | 0 | 0 |
|  | Norwegian Red | 0 | 0 | 0 | 0 | 0 | 0 | 0 | 0 | 0 | 100 | 0 | 0 | 0 | 0 | 0 | 0 |
|  | Piedmontese | 0 | 0 | 0 | 0 | 0 | 0 | 0 | 0 | 0 | 0 | 100 | 0 | 0 | 0 | 0 | 0 |
|  | Red Angus | 0 | 0 | 0 | 0 | 0 | 0 | 0 | 0 | 0 | 0 | 0 | 93.3 | 0 | 0 | 0 | 0 |
|  | Red Poll | 0 | 0 | 0 | 0 | 0 | 0 | 0 | 0 | 0 | 0 | 0 | 0 | 100 | 0 | 0 | 0 |
|  | Shorthorn | 0 | 0 | 0 | 0 | 0 | 0 | 0 | 0 | 0 | 0 | 0 | 0 | 0 | 96 | 0 | 0 |
|  | Simmental | 0 | 0 | 4 | 0 | 0 | 0 | 0 | 0 | 0 | 0 | 0 | 0 | 0 | 0 | 100 | 0 |
|  | Welsh Black | 0 | 0 | 0 | 0 | 0 | 0 | 0 | 0 | 0 | 0 | 0 | 0 | 0 | 0 | 0 | 100 |
| 200 SNPs | Angus | 100 | 0 | 0 | 0 | 0 | 0 | 0 | 0 | 0 | 0 | 0 | 0 | 0 | 0 | 0 | 0 |
|  | Brown Swiss | 0 | 100 | 0 | 0 | 0 | 0 | 0 | 0 | 0 | 0 | 0 | 0 | 0 | 0 | 0 | 0 |
|  | Charolais | 0 | 0 | 96 | 0 | 0 | 0 | 0 | 0 | 0 | 0 | 0 | 0 | 0 | 0 | 0 | 0 |
|  | Finnish Ayrshire | 0 | 0 | 0 | 100 | 0 | 0 | 0 | 0 | 0 | 0 | 0 | 0 | 0 | 0 | 0 | 0 |
|  | Guernsey | 0 | 0 | 0 | 0 | 100 | 0 | 0 | 0 | 0 | 0 | 0 | 0 | 0 | 0 | 0 | 0 |
|  | Hereford | 0 | 0 | 0 | 0 | 0 | 100 | 0 | 0 | 0 | 0 | 0 | 0 | 0 | 0 | 0 | 0 |
|  | Holstein | 0 | 0 | 0 | 0 | 0 | 0 | 100 | 0 | 0 | 0 | 0 | 0 | 0 | 0 | 0 | 0 |
|  | Jersey | 0 | 0 | 0 | 0 | 0 | 0 | 0 | 100 | 0 | 0 | 0 | 0 | 0 | 0 | 0 | 0 |
|  | Limousin | 0 | 0 | 0 | 0 | 0 | 0 | 0 | 0 | 100 | 0 | 0 | 0 | 0 | 0 | 0 | 0 |
|  | Norwegian Red | 0 | 0 | 0 | 0 | 0 | 0 | 0 | 0 | 0 | 100 | 0 | 0 | 0 | 0 | 0 | 0 |
|  | Piedmontese | 0 | 0 | 0 | 0 | 0 | 0 | 0 | 0 | 0 | 0 | 100 | 0 | 0 | 0 | 0 | 0 |
|  | Red Angus | 0 | 0 | 0 | 0 | 0 | 0 | 0 | 0 | 0 | 0 | 0 | 100 | 0 | 0 | 0 | 0 |
|  | Red Poll | 0 | 0 | 0 | 0 | 0 | 0 | 0 | 0 | 0 | 0 | 0 | 0 | 100 | 0 | 0 | 0 |
|  | Shorthorn | 0 | 0 | 0 | 0 | 0 | 0 | 0 | 0 | 0 | 0 | 0 | 0 | 0 | 100 | 0 | 0 |
|  | Simmental | 0 | 0 | 4 | 0 | 0 | 0 | 0 | 0 | 0 | 0 | 0 | 0 | 0 | 0 | 100 | 0 |
|  | Welsh Black | 0 | 0 | 0 | 0 | 0 | 0 | 0 | 0 | 0 | 0 | 0 | 0 | 0 | 0 | 0 | 100 |

Table S1a. Type I error rates for each breed following individual assignment analysis using SNP markers ranked by the pairwise Wright’s F_ST_ selection method.

| TYPE II ERRORS |  | BREED ORIGIN | | |  |  |  |  |  |  |  |  |  |  |  |  |  |
| --- | --- | --- | --- | --- | --- | --- | --- | --- | --- | --- | --- | --- | --- | --- | --- | --- | --- |
| ALLOCATED BREED |  | A | BS | CH | FA | GU | HR | HO | JR | LIM | NR | PD | RA | RP | SH | SIM | WB |
| 50 SNPs | Angus | 95.4 | 0 | 0 | 0 | 0 | 0 | 0 | 0 | 0 | 0 | 0 | 4.65 | 0 | 0 | 0 | 0 |
|  | Brown Swiss | 0 | 100 | 0 | 0 | 0 | 0 | 0 | 0 | 0 | 0 | 0 | 0 | 0 | 0 | 0 | 0 |
|  | Charolais | 0 | 0 | 85.19 | 0 | 0 | 3.7 | 7.41 | 0 | 0 | 0 | 0 | 0 | 0 | 3.7 | 0 | 0 |
|  | Finnish Ayrshire | 0 | 0 | 0 | 83.33 | 0 | 0 | 0 | 0 | 0 | 16.67 | 0 | 0 | 0 | 0 | 0 | 0 |
|  | Guernsey | 0 | 0 | 0 | 0 | 100 | 0 | 0 | 0 | 0 | 0 | 0 | 0 | 0 | 0 | 0 | 0 |
|  | Hereford | 0 | 0 | 0 | 0 | 0 | 100 | 0 | 0 | 0 | 0 | 0 | 0 | 0 | 0 | 0 | 0 |
|  | Holstein | 0 | 0 | 0 | 0 | 0 | 0 | 100 | 0 | 0 | 0 | 0 | 0 | 0 | 0 | 0 | 0 |
|  | Jersey | 0 | 0 | 0 | 0 | 0 | 0 | 0 | 100 | 0 | 0 | 0 | 0 | 0 | 0 | 0 | 0 |
|  | Limousin | 0 | 0 | 0 | 0 | 0 | 0 | 0 | 0 | 96 | 0 | 0 | 0 | 0 | 0 | 4 | 0 |
|  | Norwegian Red | 0 | 0 | 0 | 0 | 0 | 0 | 0 | 0 | 0 | 100 | 0 | 0 | 0 | 0 | 0 | 0 |
|  | Piedmontese | 0 | 0 | 0 | 0 | 4.35 | 0 | 0 | 0 | 4.35 | 0 | 82.61 | 0 | 0 | 0 | 4.35 | 4.35 |
|  | Red Angus | 35 | 0 | 0 | 0 | 0 | 0 | 0 | 0 | 0 | 0 | 0 | 65 | 0 | 0 | 0 | 0 |
|  | Red Poll | 0 | 0 | 0 | 0 | 0 | 5.26 | 0 | 0 | 0 | 0 | 0 | 0 | 94.74 | 0 | 0 | 0 |
|  | Shorthorn | 0 | 0 | 0 | 0 | 0 | 0 | 0 | 0 | 0 | 0 | 0 | 0 | 0 | 100 | 0 | 0 |
|  | Simmental | 0 | 0 | 7.69 | 0 | 0 | 0 | 0 | 0 | 0 | 0 | 0 | 0 | 0 | 3.85 | 88.46 | 0 |
|  | Welsh Black | 0 | 0 | 0 | 0 | 0 | 0 | 0 | 0 | 0 | 0 | 0 | 0 | 0 | 0 | 0 | 100 |
| 100 SNPs | Angus | 95.9 | 0 | 0 | 0 | 0 | 0 | 0 | 0 | 0 | 0 | 0 | 4.08 | 0 | 0 | 0 | 0 |
|  | Brown Swiss | 0 | 100 | 0 | 0 | 0 | 0 | 0 | 0 | 0 | 0 | 0 | 0 | 0 | 0 | 0 | 0 |
|  | Charolais | 0 | 0 | 92.31 | 0 | 0 | 0 | 0 | 0 | 0 | 0 | 0 | 0 | 0 | 3.85 | 0 | 3.85 |
|  | Finnish Ayrshire | 0 | 0 | 0 | 90 | 0 | 0 | 0 | 0 | 0 | 10 | 0 | 0 | 0 | 0 | 0 | 0 |
|  | Guernsey | 0 | 0 | 0 | 0 | 100 | 0 | 0 | 0 | 0 | 0 | 0 | 0 | 0 | 0 | 0 | 0 |
|  | Hereford | 0 | 0 | 0 | 0 | 0 | 100 | 0 | 0 | 0 | 0 | 0 | 0 | 0 | 0 | 0 | 0 |
|  | Holstein | 0 | 0 | 0 | 0 | 0 | 0 | 100 | 0 | 0 | 0 | 0 | 0 | 0 | 0 | 0 | 0 |
|  | Jersey | 0 | 0 | 0 | 0 | 0 | 0 | 0 | 100 | 0 | 0 | 0 | 0 | 0 | 0 | 0 | 0 |
|  | Limousin | 0 | 0 | 0 | 0 | 0 | 0 | 0 | 0 | 100 | 0 | 0 | 0 | 0 | 0 | 0 | 0 |
|  | Norwegian Red | 0 | 0 | 0 | 4.76 | 0 | 0 | 0 | 0 | 0 | 95.24 | 0 | 0 | 0 | 0 | 0 | 0 |
|  | Piedmontese | 0 | 0 | 0 | 0 | 0 | 0 | 0 | 0 | 0 | 0 | 100 | 0 | 0 | 0 | 0 | 0 |
|  | Red Angus | 7.14 | 0 | 0 | 0 | 0 | 0 | 0 | 0 | 0 | 0 | 0 | 92.86 | 0 | 0 | 0 | 0 |
|  | Red Poll | 0 | 0 | 0 | 0 | 0 | 0 | 0 | 0 | 0 | 0 | 0 | 0 | 100 | 0 | 0 | 0 |
|  | Shorthorn | 0 | 0 | 0 | 0 | 0 | 0 | 0 | 0 | 0 | 0 | 0 | 0 | 0 | 100 | 0 | 0 |
|  | Simmental | 0 | 0 | 3.57 | 0 | 0 | 0 | 0 | 0 | 3.57 | 0 | 0 | 0 | 0 | 3.57 | 89.29 | 0 |
|  | Welsh Black | 0 | 0 | 0 | 0 | 0 | 0 | 0 | 0 | 0 | 0 | 0 | 0 | 0 | 0 | 0 | 100 |
| 150 SNPs | Angus | 98 | 0 | 0 | 0 | 0 | 0 | 0 | 0 | 0 | 0 | 0 | 2.04 | 0 | 0 | 0 | 0 |
|  | Brown Swiss | 0 | 100 | 0 | 0 | 0 | 0 | 0 | 0 | 0 | 0 | 0 | 0 | 0 | 0 | 0 | 0 |
|  | Charolais | 0 | 0 | 96 | 0 | 0 | 0 | 0 | 0 | 0 | 0 | 0 | 0 | 0 | 4 | 0 | 0 |
|  | Finnish Ayrshire | 0 | 0 | 0 | 100 | 0 | 0 | 0 | 0 | 0 | 0 | 0 | 0 | 0 | 0 | 0 | 0 |
|  | Guernsey | 0 | 0 | 0 | 0 | 100 | 0 | 0 | 0 | 0 | 0 | 0 | 0 | 0 | 0 | 0 | 0 |
|  | Hereford | 0 | 0 | 0 | 0 | 0 | 100 | 0 | 0 | 0 | 0 | 0 | 0 | 0 | 0 | 0 | 0 |
|  | Holstein | 0 | 0 | 0 | 0 | 0 | 0 | 100 | 0 | 0 | 0 | 0 | 0 | 0 | 0 | 0 | 0 |
|  | Jersey | 0 | 0 | 0 | 0 | 0 | 0 | 0 | 100 | 0 | 0 | 0 | 0 | 0 | 0 | 0 | 0 |
|  | Limousin | 0 | 0 | 0 | 0 | 0 | 0 | 0 | 0 | 100 | 0 | 0 | 0 | 0 | 0 | 0 | 0 |
|  | Norwegian Red | 0 | 0 | 0 | 0 | 0 | 0 | 0 | 0 | 0 | 100 | 0 | 0 | 0 | 0 | 0 | 0 |
|  | Piedmontese | 0 | 0 | 0 | 0 | 0 | 0 | 0 | 0 | 0 | 0 | 100 | 0 | 0 | 0 | 0 | 0 |
|  | Red Angus | 0 | 0 | 0 | 0 | 0 | 0 | 0 | 0 | 0 | 0 | 0 | 100 | 0 | 0 | 0 | 0 |
|  | Red Poll | 0 | 0 | 0 | 0 | 0 | 0 | 0 | 0 | 0 | 0 | 0 | 0 | 100 | 0 | 0 | 0 |
|  | Shorthorn | 0 | 0 | 0 | 0 | 0 | 0 | 0 | 0 | 0 | 0 | 0 | 0 | 0 | 100 | 0 | 0 |
|  | Simmental | 0 | 0 | 3.85 | 0 | 0 | 0 | 0 | 0 | 0 | 0 | 0 | 0 | 0 | 0 | 96.15 | 0 |
|  | Welsh Black | 0 | 0 | 0 | 0 | 0 | 0 | 0 | 0 | 0 | 0 | 0 | 0 | 0 | 0 | 0 | 100 |
| 200 SNPs | Angus | 100 | 0 | 0 | 0 | 0 | 0 | 0 | 0 | 0 | 0 | 0 | 0 | 0 | 0 | 0 | 0 |
|  | Brown Swiss | 0 | 100 | 0 | 0 | 0 | 0 | 0 | 0 | 0 | 0 | 0 | 0 | 0 | 0 | 0 | 0 |
|  | Charolais | 0 | 0 | 100 | 0 | 0 | 0 | 0 | 0 | 0 | 0 | 0 | 0 | 0 | 0 | 0 | 0 |
|  | Finnish Ayrshire | 0 | 0 | 0 | 100 | 0 | 0 | 0 | 0 | 0 | 0 | 0 | 0 | 0 | 0 | 0 | 0 |
|  | Guernsey | 0 | 0 | 0 | 0 | 100 | 0 | 0 | 0 | 0 | 0 | 0 | 0 | 0 | 0 | 0 | 0 |
|  | Hereford | 0 | 0 | 0 | 0 | 0 | 100 | 0 | 0 | 0 | 0 | 0 | 0 | 0 | 0 | 0 | 0 |
|  | Holstein | 0 | 0 | 0 | 0 | 0 | 0 | 100 | 0 | 0 | 0 | 0 | 0 | 0 | 0 | 0 | 0 |
|  | Jersey | 0 | 0 | 0 | 0 | 0 | 0 | 0 | 100 | 0 | 0 | 0 | 0 | 0 | 0 | 0 | 0 |
|  | Limousin | 0 | 0 | 0 | 0 | 0 | 0 | 0 | 0 | 100 | 0 | 0 | 0 | 0 | 0 | 0 | 0 |
|  | Norwegian Red | 0 | 0 | 0 | 0 | 0 | 0 | 0 | 0 | 0 | 100 | 0 | 0 | 0 | 0 | 0 | 0 |
|  | Piedmontese | 0 | 0 | 0 | 0 | 0 | 0 | 0 | 0 | 0 | 0 | 100 | 0 | 0 | 0 | 0 | 0 |
|  | Red Angus | 0 | 0 | 0 | 0 | 0 | 0 | 0 | 0 | 0 | 0 | 0 | 100 | 0 | 0 | 0 | 0 |
|  | Red Poll | 0 | 0 | 0 | 0 | 0 | 0 | 0 | 0 | 0 | 0 | 0 | 0 | 100 | 0 | 0 | 0 |
|  | Shorthorn | 0 | 0 | 0 | 0 | 0 | 0 | 0 | 0 | 0 | 0 | 0 | 0 | 0 | 100 | 0 | 0 |
|  | Simmental | 0 | 0 | 3.85 | 0 | 0 | 0 | 0 | 0 | 0 | 0 | 0 | 0 | 0 | 0 | 96.15 | 0 |
|  | Welsh Black | 0 | 0 | 0 | 0 | 0 | 0 | 0 | 0 | 0 | 0 | 0 | 0 | 0 | 0 | 0 | 100 |

Table S1b. Type II error rates for each breed following individual assignment analysis using SNP markers ranked by the pairwise Wright’s F_ST_ selection method


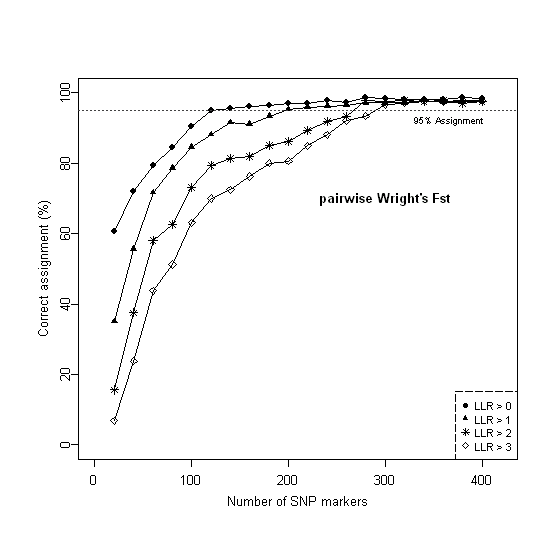


Fig S2. Cross-validation individual assignment. Based on allele frequencies of ‘training set’ (after removal of ‘hold-out’ individuals) and assignment of ‘hold-out’ individuals to breed origin. The percentage assignment success with cumulative number of top-ranked SNP markers at the 4 stringency threshold levels, for pairwise Wright’s F_ST_ selection method.
